# Effect of OTX-101 0.09% ciclosporin solution on clinical signs of dry eye disease in patients with moderate-to-severe corneal damage: a post-hoc analysis of randomized clinical trials

**DOI:** 10.1007/s00417-025-07054-7

**Published:** 2025-12-20

**Authors:** Maurizio Rolando, José Manuel Benitez del Castillo, Maria Antonia Cebollero, Elisabeth M. Messmer

**Affiliations:** 1 Centro Superficie Oculare ISPRE OFTALMICA srl, Genova, 16129 Italy; 2 Department of Ophthalmology, Ocumed-Clínica Rementería, Hospital Clínico San Carlos, Universidad Complutense de Madrid, Madrid, Spain; 3 Sun Pharmaceutical Industries B. V., Hoofddorp, The Netherlands; 4 Department of Ophthalmology, Ludwig Maximilians University, Munich, Germany

**Keywords:** Cyclosporine, Ophthalmic solution, Keratoconjunctivitis sicca

## Abstract

**Purpose:**

This post-hoc analysis of two OTX-101 clinical trials assessed the efficacy of 0.09% ciclosporin in treating dry eye disease (DED) patients with moderate-to-severe corneal damage.

**Methods:**

The criteria for moderate-to-severe corneal damage were defined as a CFS total score (5 zones) of ≥ 6 or single zone ≥ 2 at baseline (modified NEI scale). 516 patients (49% of the intention-to-treat population) met these criteria at baseline. Changes in ocular surface damage (cornea and conjunctiva) and aqueous tear deficiency from baseline were determined through corneal fluorescein staining (CSF), Lissamine green staining (LGS) and Schirmer’s test without anesthesia (ST).

**Results:**

After 12 weeks of treatment with OTX-101 versus (vs.) vehicle, outcomes improved across six parameters: (I) Central CFS mean change from baseline was -0.48 vs. -0.36, *p* = 0.0144. (II) Total CFS corneal staining change from baseline was -2.30 vs. -1.61 *p* = 0.0004. (III) Improvement ≥ 50% in total CFS from baseline was 44.8% vs. 33.4% *p* = 0. 0010. (IV) Conjunctival damage as measured by LGS mean change from baseline was -1.61 vs. -0.99, *p* = 0.0006. (V) Tear production as measured by ST as percentage of eyes with ≥ 10 mm increase from baseline was 18.2% vs. 8.3% *p* = 0.0003, and (VI) ST mean change from baseline was +2.94 vs. +0.28 *p* = 0.0008.

**Conclusion:**

Administration of OTX-101 0.09% leads to statistically significant improvement across six parameters in ocular surface staining and tear production in DED patients with moderate to severe corneal damage.

## Abstract (highlights)

**Key messages:**

*****What is known***:**

- OTX-101 0.09% CsA is a nanomicellar, aqueous formulation of ciclosporin A, designed to enhance ocular bioavailability and therapeutic efficacy for treating DED. The safety and efficacy of OTX-101 0.09% CsA have been validated in two randomized, double-masked, vehicle-controlled clinical trials.

*****What is new***:**

- This post-hoc analysis of pooled data from the two clinical trials focused on patients with moderate-to-severe corneal damage. In this subgroup, treatment with OTX-101 0.09% had a statistically significant benefit over vehicle across three clinical measures: Corneal Fluorescein Staining, Lissamine Green Staining and Schirmer’s Test.

## Introduction

Dry eye disease (DED) is a multifactorial disorder causing deficiencies in tear production, loss of tear film homeostasis with tear film instability, hyperosmolarity, irritation and inflammation of the cornea and conjunctiva, and concomitant damage to the ocular surface [1–4]. It leads to ocular surface damage, resulting in mild to severe discomfort and sensation of stinging, grittiness and visual disturbance [4–6]. In this study, subgroups of patients with moderate to severe corneal damage were identified from two clinical trials and evaluated across parameters of aqueous deficiency, measured by Schirmer’s test (ST), corneal damage, assessed through Corneal Fluorescein Staining (CFS) [5], and conjunctival damage, measured through Lissamine Green Staining (LGS) [6].

Ciclosporin (CsA) is a potent immunomodulatory agent that targets the inflammatory response in DED. However, due to its hydrophobic nature, ocular penetration is limited, thus leading to challenges in delivery to the ocular surface. Oil-in-water emulsions of ciclosporin can cause blurred vision, irritation, and delayed onset of efficacy due to low bioavailability and tissue penetration. A novel ciclosporin formulation should enhance ocular bioavailability, provide better therapeutic effects, and reduce adverse effects [7]. OTX-101 0.09% (Cequa^®^) is a nanomicellar CsA solution approved in the U.S. for increasing tear production in DED [8], and in the EU with the indication of treating moderate to severe DED. OTX-101 utilizes nanomicellar technology, in which amphiphilic polymers self-assemble into micelles, encapsulating the hydrophobic ciclosporin A within a hydrophilic outer coat. This enhances drug solubility, ocular tissue penetration, and bioavailability [7, 8].

Two previous clinical trials of OTX-101 demonstrated that 12 weeks of daily delivery to the ocular surface significantly improved tear production while reducing conjunctival and corneal damage as evidenced by CFS and LGS [9, 10]. In these studies, adverse effects were mainly mild to moderate. A third trial established the long-term safety of OTX-101, supporting its continued use in clinical practice for improving tear production and ocular surface health [11]. The objective of the current study is a post hoc analysis of the two similarly designed OTX-101 clinical trials (OTX-101–2014-001 phase IIb/III and OTX-101–2016-001 phase III; see methods) [9, 10] by focusing on a subgroup of DED patients having moderate-to-severe corneal damage. This was based on the rationale that the two former trials included many patients with mild symptoms and the effectiveness of 0.09% ciclosporin should be statistically analyzed in a more severely affected group. To identify patients with moderate-to-severe corneal damage within the two clinical trial cohorts, the criteria based on CFS scores were defined (see methods) and used to determine the subgroup that is the subject of this post hoc analysis.

## Methods

### Establishing a post-hoc analysis intention-to-treat (ITT) population

This study established the ITT population from OTX-101–2014-001 phase IIb/III [10] and OTX-101–2016-001 phase III [9] clinical trial cohorts previously treated and analyzed. Both clinical trials were double-masked, randomized, vehicle-controlled and evaluated the safety and efficacy of OTX-101. Both had identical inclusion and exclusion criteria and the patient populations were defined by similar DED severity, e.g. Lissamine green staining score of 3 to 9 and a global symptom score ≥ 40. Also, both were assessed for similar endpoints using the same standardized ophthalmic tests (e.g., conjunctival LGS, ST without anesthesia, and CFS). Patients participated for approximately 100 days, including a run-in-period of 14 days of vehicle application to wash out any previously used topical medications and a treatment period of 12 weeks/84 days. Patients were instructed to apply one drop of the ophthalmic formulation twice daily, approximately 12 h apart. Key inclusion criteria in these studies were: adult patients with ≥ 6-month history of DED and diagnosis of bilateral DED at screening, and total conjunctival staining score of 3 to 9 in the same eye at screening and baseline (NEI scale). Exclusion criteria were use of ciclosporin ophthalmic emulsion 0.05% within 3 months before screening, prior treatment failure of ciclosporin ophthalmic emulsion 0.05%, and a diagnosis of Sjögren’s disease at least 5 years before the screening visit.

To establish the ITT population for this post hoc analysis patient data from both clinical trial cohorts were pooled. The phase III analysis assessed efficacy endpoints bilaterally, capturing data from both eyes for comparisons (average eye) [9]. The OTX-101 phase IIb/III clinical dataset established worse eye and average eye data, while only worse eye data were published [10]. Because that trial evaluated ciclosporin at 0.09% and 0.05%, patients who had received the lower concentration were excluded from this analysis. Finally, the criteria outlined in the next two sections were applied to identify patients with moderate-to-severe disease.

### Testing methods 

Corneal Fluorescein Staining (CFS): measured staining in five corneal regions (central, superior, inferior, nasal, temporal). Each region was scored with half-point increments on a 0 to 4 scale, with 0 = no staining, and 4 = severe diffuse staining with coalescent lesions (modified NEI scale) [12]. Lissamine Green Staining (LGS): evaluated staining of conjunctiva with a total score range of 0 to 12. LGS was assessed in four conjunctival regions: temporal, nasal, two inferior zones. Measurements of two superior zones were excluded from the total score for analysis. This was an effort to reduce variability across the treatment groups because the superior areas are usually protected from dryness and cell damage by the eye lids. Each region was scored on a 0 to 3 scale, with 0 = no staining, and 3 = dense confluent staining. Schirmer’s Tear Test (ST): assessed tear production by measuring strip wetting over 5 min (without anesthesia).

### Defining the moderate-to-severe corneal damage patient subgroup

The modified NEI (National Eye Institute)/Industry Workshop grading scale for Corneal Fluorescein Staining was used to define the subgroup [12]. According to this the five corneal zones were individually scored at baseline, from 0 (no stain) to 4 (severe stain) to then calculate the NEI score by summing. A total score of ≥ 6 or a score ≥ 2 for a single zone was taken as a criterium for moderate-to-severe corneal damage.

### Testing parameters and endpoints

The following test results were established at treatment day 84: I: Central CFS mean change from baseline (average of eyes); II: total CFS change from baseline (average of eyes); III: ≥ 50% total CFS improvement from baseline (% of eyes of both eyes); IV: change from baseline in Lissamine green staining (average of eyes); V: % of eyes with Schirmer’s test ≥ 10 mm increase from baseline (% of eyes of both eyes); VI: Schirmer’s test score mean change from baseline (average of eyes). A comparison was performed between OTX-101 (0.09%) and vehicle at day 84 for the endpoints.

### Statistics and endpoint description

Statistical analysis was conducted to evaluate changes in Schirmer’s score, Lissamine green staining, and corneal staining measures. The percentage of eyes with a ≥ 10 mm increase in Schirmer’s score was evaluated using a generalized estimating equation (GEE). The mean change from baseline in Schirmer’s score was assessed using analysis of covariance (ANCOVA), following the primary analysis of the phase III study [9]. Changes in LGS were analyzed using a mixed model for repeated measurements (MMRM), while central corneal staining (CFS) and total CFS were evaluated using ANCOVA. The percentage of eyes achieving ≥ 50% improvement in total corneal staining was determined based on relative change from baseline, analyzed using GEE. To account for multiple comparisons, Hochberg-adjusted p-values were applied to maintain statistical accuracy.

## Results

### Patients

Three hundred and four patients of the phase IIb/III clinical trial [10] and all 744 randomized patients from the phase III trial [9] were pooled to establish an ITT population. Based on corneal fluorescein staining, a subgroup of patients with moderate to severe corneal damage was established at baseline. The critical parameters were defined as a CFS total score (5 zones) of ≥ 6 or single zone ≥ 2 at baseline (modified NEI scale). This subset included 516 patients with moderate to severe corneal damage. The patients were randomized into treatment and vehicle groups, with 259 subjects in the active treatment, and 257 subjects in the vehicle arm to be analyzed for day 84 of treatment.

### Corneal staining in patients with moderate-to-severe corneal damage

**Fig. 1:**
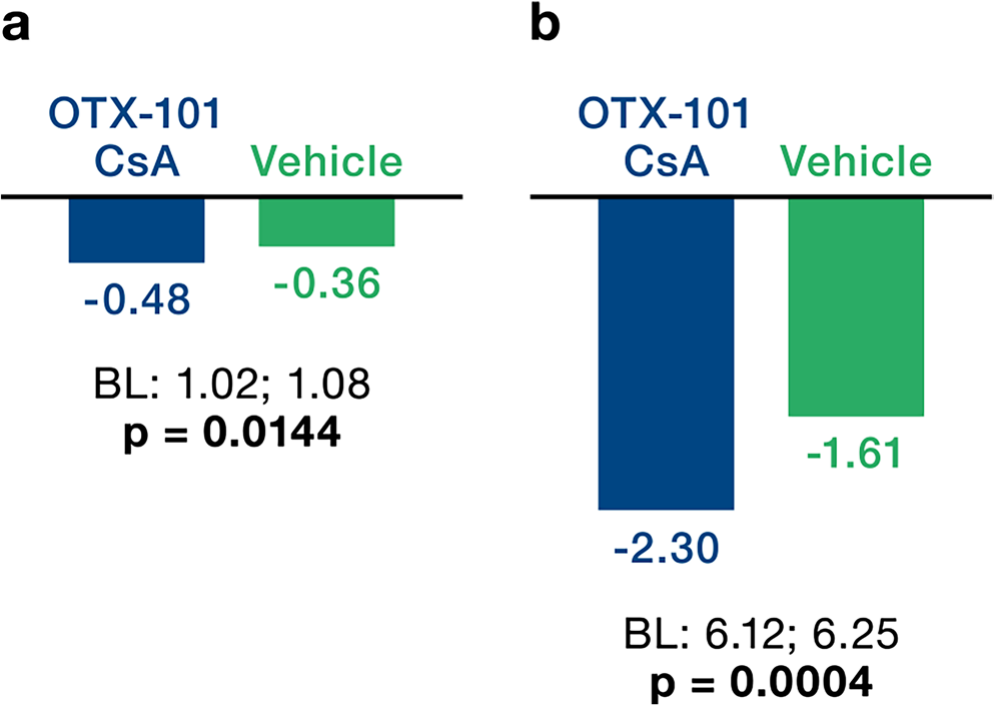
(A) Central corneal fluorescein staining change from baseline (mean Δ at day 84). (B) Total corneal fluorescein staining change from baseline (mean Δ at day 84). Hochberg adjusted p-values

**Fig. 2:**
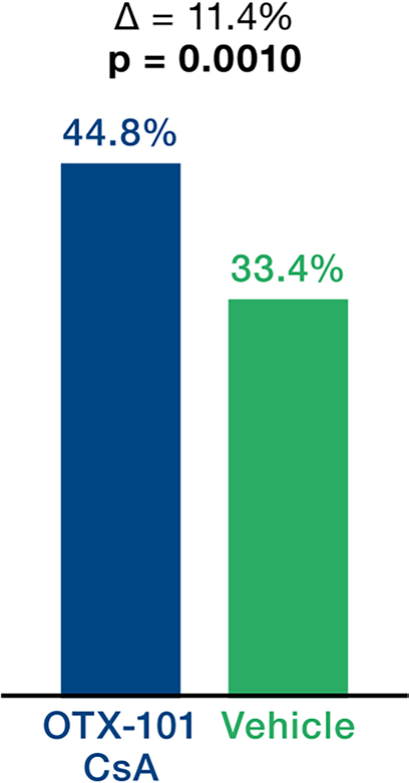
Total corneal staining change from baseline (mean Δ at day 84). Hochberg adjusted p-values

The cornea was evaluated by corneal fluorescein staining. The endpoints established included the mean change in central CFS mean change from baseline, total CFS change from baseline, and a ≥ 50% total CFS change from baseline. The CFS showed statistically significant therapeutic effects (Fig. 1A and B). The change in central CFS from baseline was -0.48 for OTX-101 compared to -0.36 for vehicle (*p* = 0.0144; Fig. 1A), and total CFS changed by -2.30 for OTX-101 versus -1.61 for the vehicle (*p* = 0.0004; Fig. 1B), indicating a significant effect of OTX-101 in alleviating corneal damage. A clinically meaningful improvement of ≥ 50% total CFS from baseline was observed in 44.8% of eyes, compared to 33.4% at day 84 (*p* = 0.0010; Fig. 2).

Thus, OTX-101 had a measurable and significant effect across all three measurements of corneal damage.

#### Analysis of conjunctival damage in patients with moderate-to-severe corneal damage

Lissamine Green Staining was used to assess conjunctival damage. The dye selectively stains devitalized and apoptotic cells on the ocular surface, making this technique particularly useful for evaluating the extent of conjunctival epithelial disruption.

**Fig. 3:**
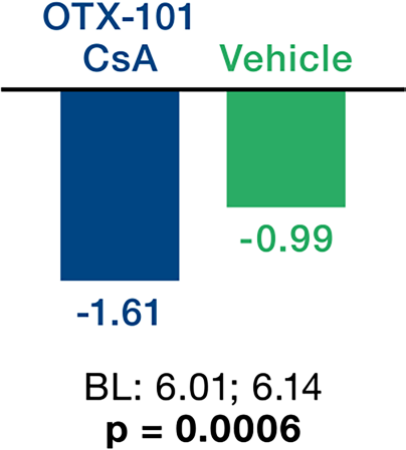
Lissamine Green Staining: Score change from baseline in sum of non-superior zones (4-zone) scores (mean Δ at day 84). Hochberg adjusted p-values

The total score ranged from 0 to 12 (data not shown). By day 84, the mean change in LGS score in the sum of non-superior zones was -1.61 for OTX-101 versus -0.99 for the vehicle, demonstrating a significant reduction in conjunctival staining (*p* = 0.0006; Fig. 3).

Thus, the damage to the conjunctiva, as measured by LGS, was significantly improved after treatment for 84 days.

#### Analysis of tear production in patients with moderate-to-severe corneal damage

**Fig. 4:**
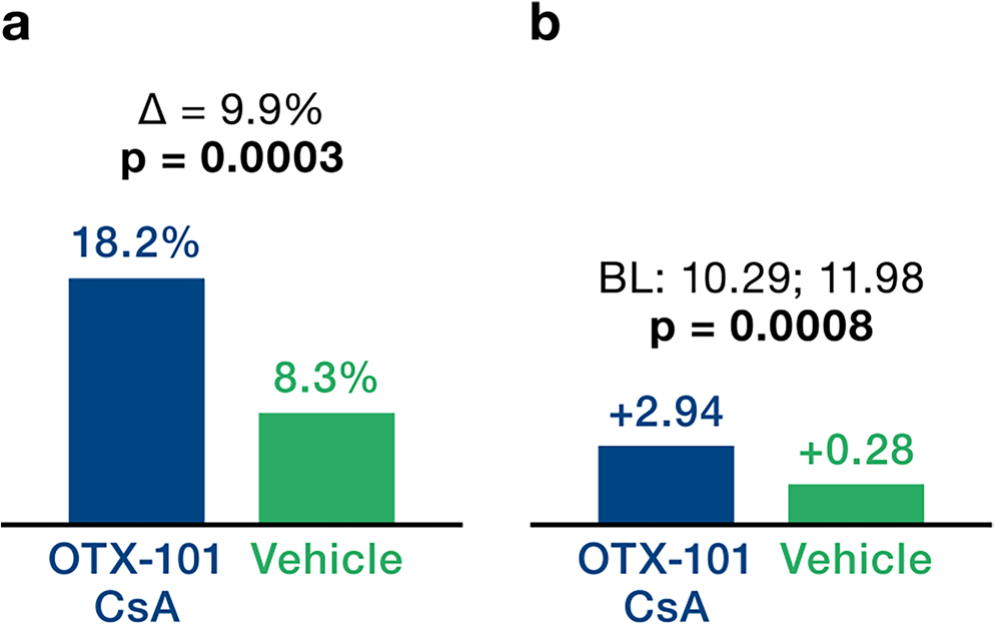
(A) Schirmer’s Tear Test: Percent of eyes with ≥ 10 mm increase from baseline (% of eyes at day 84). (B) Score change from baseline (mean Δ at day 84). Hochberg adjusted p-values

Schirmer’s Tear Test provides quantitative data on tear secretion by assessing the wetting length of a standardized filter paper strip over 5 min. Two endpoints were analyzed. By day 84, 18.2% of eyes in the OTX-101 group exhibited a ≥ 10 mm increase in tear production from baseline, compared to 8.3% in the vehicle group (*p* = 0.0003; Fig. 4A).

The mean change in Schirmer’s score change at day 84 from baseline (measured as average of eyes) was + 2.94 for OTX-101 versus + 0.28 for the vehicle (*p* = 0.0008; Fig. 4B).

These results demonstrate that tear production significantly increased after treatment with OTX-101.

This post hoc analysis of a subpopulation of patients with moderate to severe corneal damage demonstrated statistically significant effects of OTX-101 0.09% treatment versus vehicle across all three tests (CFS, LGS, and Schirmer’s). It had measurable and significant effects on all six endpoints and ameliorated clinically measurable aspects of moderate-to severe DED after 12 weeks.

## Discussion

OTX-101 is a novel formulation of ciclosporin via nanomicelles that results in a clear solution rather than a suspension, aiding in its delivery to the ocular surface [8]. This post-hoc study comprehensively evaluated how OTX-101 affects both tear production and ocular surface integrity in patients with moderate-to-severe corneal damage by analyzing a patient population that was determined from two previous OTX-101 clinical trials [9, 10]. It showed a statistically significant improvement in dry eye disease (DED) signs through topical application of OTX-101 0.09% treatment compared to the vehicle, a treatment effect consistent across Schirmer’s test, corneal fluorescein staining, and Lissamine green staining. Corneal fluorescein staining serves as a crucial indicator of ocular surface damage. As noted by Rolando et al. (2023) [13], corneal damage from chronic inflammation leads to tear film instability, oxidative stress, and epithelial cell dysfunction, and in consequence impairs vision quality. Here, we demonstrate a statistically significant reduction in central and total corneal staining. This suggests that OTX-101 improves corneal epithelial integrity and improves ocular surface smoothness, which contributes to better visual acuity and reduced eye irritation. Lissamine green staining is not only a marker of DED severity, it also indicates conjunctival damage that reflects ongoing inflammation, particularly T-cell-mediated immune responses [14]. It points to inflammatory damage of the ocular surface, particularly goblet cell loss and mucin deficiency, which compromise tear film stability and lubrication. The established significant reduction in LGS further adds to the role of OTX-101 in restoring ocular surface homeostasis by modulating inflammation.

This analysis demonstrated that OTX-101 0.09% significantly improved corneal as well as conjunctival DED signs in a subpopulation of patients with moderate-to-severe corneal damage. Further, tear production of the affected eyes was found significantly elevated, a finding corroborated by two previous pooled analyses of the same clinical trials [15, 16]. Toyos et al. [15] specifically focused on a subgroup of patients with severe tear deficiency (Schirmer’s score < 5 mm at baseline) and demonstrated a significant increase in tear production at day 84 (mean increase of + 5.5 mm) with 22.6% of patients achieving a ≥ 10 mm increase in Schirmer’s score compared to 10.6% in the vehicle group (*p* = 0.0168). This suggests that patients with severe aqueous deficiency may experience the greatest response to treatment. In contrast, focusing on moderate-to-severe corneal damage, patients had a more modest yet statistically significant increase in Schirmer’s scores (+ 2.94 mm, *p* = 0.0008). Sheppard et al. [15] assessed the ITT population and a subgroup of patients with Schirmer’s score < 10 mm at baseline. In the ITT population, 16.6% of eyes receiving OTX-101 achieved a ≥ 10 mm increase in Schirmer test scores, compared to 9% in the vehicle group (*p* < 0.0001). In the < 10 mm subgroup, 18.7% of OTX-101-treated eyes met this threshold, compared to 10.2% in the vehicle group (*p* = 0.0001), demonstrating a statistically significant improvement in tear production. The findings from these two studies [15, 16] show that OTX-101 enhances tear production in patients with severe aqueous deficiency.

This post-hoc analysis expands on these findings by demonstrating improvements in ocular surface integrity, as evidenced by significantly reduced corneal fluorescein stainings (*p* = 0.0004) and conjunctival Lissamine green stainings (*p* = 0.0006). It further emphasizes that the benefits of OTX-101 extend beyond tear production, providing measurable enhancements in ocular surface health in a broader DED population. As the clinical trials have evaluated multiple endpoints, this additional evidence affirms the role of OTX-101 in restoring ocular surface integrity, which is essential for the long-term management of DED beyond merely relieving symptoms. In fact, a European consensus was established through a panel of seven experts on the anti-inflammatory treatment of DED, providing a thorough practical guideline on how cyclosporin is applied for proper DED management [14]. It is worth discussing the fact that earlier clinical trials also tested a lower ciclosporin concentration (0.05%) [9] but only the 0.09% nanomicellar solution proceeded to gain market access. The reason was a higher effectiveness of the 0.09% solution. A recently published multicenter, randomized, double-masked trial with 450 DED patients compared the two treatments, each applied twice daily for three months [17]. Compared to 0.05% ciclosporin, treatment with the 0.09% solution led to significantly greater improvements in tear film osmolarity, Lissamine green and corneal fluorescein staining, and Schirmer test scores compared to 0.05%. However, the symptom score improvements did not differ significantly between the two concentrations. Both groups experienced similar side effects, mainly burning, pain, and redness, typically rated as severe, although adverse events were more frequent with 0.09% (17.8% versus 9.7%). Initial discomfort was higher in the 0.09% group but improved over time, and overall comfort across the treatment period was significantly better with 0.05%.

Generally, clinical trials on OTX-101 have found that it is well tolerated, with adverse effects typically limited to mild burning or stinging after topical application of the drug [9–11]. Adverse reactions were summarized from pooled phase IIb/III and phase III trial analyses; the most frequently reported adverse reactions were instillation site pain (21.6% versus 3.8% in the vehicle group), conjunctival hyperaemia (5.7% versus 3.6% in the vehicle group), and eye irritation (1.1% versus 0.2% in the vehicle group). The long-term safety of OTX-101 was evaluated in a one-year extension of a phase 3 efficacy trial. The most common adverse event was instillation site pain, which was mild to moderate in severity [11].

## Conclusion

This post hoc analysis of two clinical trials demonstrates that OTX-101 0.09% significantly improves both tear production and ocular surface integrity in a more severely affected DED patient population than previously statistically assessed. Corneal and conjunctival staining, along with Schirmer’s test scores served as endpoints and show that assessing both tear function and ocular surface health is important when evaluating treatment efficacy. This analysis confirms the role of OTX-101 0.09% as a comprehensive therapy for DED patients with moderate-to-severe corneal damage.
